# Effect of two types of shoulder prosthesis on the muscle forces using a generic multibody model for different arm motions

**DOI:** 10.1186/s12938-022-00988-7

**Published:** 2022-03-19

**Authors:** Bernhard Weisse, Susan Lama, Gabor Piskoty, Christian Affolter, Ameet K. Aiyangar

**Affiliations:** Laboratory for Mechanical Systems Engineering, Empa, Swiss Federal Laboratories for Materials Science and Technology, CH-8600 Dübendorf, Switzerland

**Keywords:** Reverse shoulder prosthesis, Dual-bearing glenoid-sparing shoulder prosthesis, Musculoskeletal model, OpenSim, shoulder biomechanics, Muscle activation

## Abstract

**Background:**

This study aims to analyze the effects of a novel dual-bearing shoulder prosthesis and a conventional reverse shoulder prosthesis on the deltoid and rotator cuff muscle forces for four different arm motions. The dual-bearing prosthesis is a glenoid-sparing joint replacement with a moving center of rotation. It has been developed to treat rotator cuff arthropathy, providing an increased post-operative functionality.

**Methods:**

A three-dimensional musculoskeletal OpenSim® model of an upper body, incorporating a natural gleno-humeral joint and a scapula-thoracic joint developed by Blana et al. (J Biomech 41: 1714-1721, 2008), was used as a reference for the natural shoulder. It was modified by integrating first a novel dual-bearing prosthesis, and second, a reverse shoulder prosthesis into the shoulder joint complex. Four different arm motions, namely abduction, scaption, internal and external rotation, were simulated using an inverse kinematics approach. For each of the three models, shoulder muscle forces and joint reaction forces were calculated with a 2 kg weight in the hand.

**Results:**

In general, the maximal shoulder muscle force and joint reaction force values were in a similar range for both prosthesis models during all four motions. The maximal deltoid muscle forces in the model with the dual-bearing prosthesis were 18% lower for abduction and 3% higher for scaption compared to the natural shoulder. The maximal rotator cuff muscle forces in the model with the dual-bearing prosthesis were 36% lower for abduction and 1% higher for scaption compared to the natural shoulder. Although the maximal deltoid muscle forces in the model with the dual-bearing prosthesis in internal and external rotation were 52% and 64% higher, respectively, compared to the natural shoulder, the maximal rotator cuff muscle forces were 27% lower in both motions.

**Conclusion:**

The study shows that the dual-bearing shoulder prosthesis is a feasible option for patients with rotator cuff tear and has a strong potential to be used as secondary as well as primary joint replacement. The study also demonstrates that computer simulations can help to guide the continued optimization of this particular design concept for successful clinical outcomes.

## Background

Total shoulder arthroplasty is broadly classified into two categories: anatomical total shoulder arthroplasty and reverse total shoulder arthroplasty. Anatomical total shoulder arthroplasty retains the orientation of the natural joint and involves replacing the gleno-humeral joint with an anatomical shoulder prosthesis (ASP), wherein the natural humeral head is replaced by a ceramic or highly polished metal ball connected to the humerus by a stem. The ball head articulates with a concave polyethylene (PE) component, which is fixed onto the glenoid surface. Damage to the polyethylene glenoid component of the ASP and loosening of its fixation are the most common modes of failure [1–3].

With the overall goal of reducing the high failure rates of the "conventional" ASP, P. Grammont developed in 1985 the reverse shoulder prosthesis, wherein the ball head is fixed in the glenoid cavity instead of the humerus, while the PE cup is implanted into the humerus [4–6]. Both, the anatomical and reverse prosthetic designs are based on the assumption that the gleno-humeral joint is well represented by a purely 3 degree-of-freedom rotational joint with a fixed center of rotation. Nevertheless, during reverse arthroplasty surgery, the medialized center of rotation (COR) of the artificial gleno-humeral joint is altered by setting it in a more medial location compared to the natural shoulder joint. This modification is intended to increase the contribution of the deltoid muscles to large shoulder movements such as abduction and scaption to the reaction moment at the shoulder without substantially increasing muscle effort. This is accomplished by increasing the deltoid's lever arm by up to 42%, thus reducing deltoid muscle forces without significantly compromising the torque generated by these muscles to drive functional movements such as scaption [7, 8]. This solution is particularly beneficial when massive tears or other forms of damage to the rotator cuff muscles (e.g., supraspinatus) significantly reduce the otherwise substantial contribution of this muscle group to large shoulder movements and overall shoulder stability. The worldwide acceptance of RSP attests to its efficacy [9], and indications for reverse shoulder arthroplasty have been expanded to all forms of gleno-humeral joint disease with rheumatoid arthritis and serious rotator cuff tears, presently accounting for more than 40% of the shoulder arthroplasty market [10, 11].

The growing popularity of reverse shoulder arthroplasty notwithstanding [12–14], this surgical intervention is also associated with some significant disadvantages. The complication rate for primary arthroplasty is around 24%, which increases to 40% with revision surgeries [15, 16]. Of these, glenoid component loosening is the weak link in shoulder replacement, accounting for nearly one-third of all TSA complications [17]. Outright implant failure rates at 5- and 10-years are 10% and 22%, respectively [18], which is high compared to knee and hip replacements [19–21]. Second, patients affected by these complications must undergo revision surgeries. However, for those with a compromised bone stock (from osteolysis or bone loss due to additional preparation requirements following removal of primary prosthesis fixation), no reliable solution is currently available on the market and arthrodesis, although sub-optimal, is the final solution. A third limitation pertains to the design based on a fixed COR and no translational degrees of freedom common to both anatomical and reverse shoulder prostheses. Recent dynamic radiographic imaging studies seem to indicate otherwise [22–24]. More pertinently, while a fixed COR may simplify design and manufacturing of the prosthesis and provide some advantages for arm elevation motion, it requires a precise implantation by the surgeon and may compromise certain muscle lever-arms for other shoulder movements crucial for independent daily living.

In an attempt to address these limitations, a new shoulder prosthesis with a novel dual-bearing design, the so-called dual-bearing shoulder prosthesis (DBSP), has recently been developed and patented by the Swiss company *41hemiverse AG* [25, 26]. One major advantage is that the prosthesis can be implanted with a less invasive surgical procedure compared to conventional shoulder prostheses. In addition, the proximal component does not need a fixation to the glenoid, which represents a benefit for patients with compromised bone stock, especially at revision surgery. Furthermore, the DBSP with two, eccentrically located rotational centers partially simulates a moving COR, thus theoretically providing an improved replication of natural shoulder gleno-humeral joint kinematics.

Our overall goal is to assess the biomechanical performance of this new and promising prosthesis design and, eventually, to assess its performance in vivo and in comparison with existing solutions.

An ideal scientific study would be to implant the DBSP into patients and compare performance and outcome with conventional designs. However, from an ethical viewpoint, it is necessary to first make preliminary assessments to verify the claims with respect to superior (or at the very least equivalent) biomechanical performance compared to the existing solutions. Furthermore, measuring in vivo joint or muscle loads to assess biomechanical performance is not readily feasible, and computational musculoskeletal modeling and simulation is a valuable technique to analyze the influence of a prosthesis on the muscle forces in silico. Hence, in a first step, our study focuses on comparing the computed joint reaction forces as well as the demand on the muscles when using the novel implant with the conventional counterparts, since this is often an accepted design criterion as well as a criterion for determining placement of the implant by the surgeon during surgery.

Several studies have shown the power of 3D dynamic rigid multibody simulation tools for determining movements and internal loads within the musculoskeletal system. Opensim® is one of the software suited for simulating and predicting muscle forces and also contains a repository of models developed by research groups worldwide. The software was developed by the National Centers for Biomedical Computing at Stanford University and is an established open source software [27]. This tool was chosen because many studies demonstrated its usability for shoulder movement analysis [10, 13, 28–33] and it also offers validated basic models which can be adapted for comparative investigations. The main objective of the current study was to assess the influence of the new DBSP design on the muscle forces and joint reaction forces for four different motions. Additionally, the muscles forces and joint reaction forces estimated for the novel DBSP prosthesis in question were compared with models representing the more conventional, but widely accepted RSP, as well as the natural shoulder (NS).

## Results

Peak reaction forces at the joint, as well as peak forces generated in the individual shoulder muscles during four different movements were assessed in each of the three musculoskeletal models: NS, DBSP, and RSP model (Table 1). Of these, two are large range-of-motion (ROM) movements—abduction 0°–120°; and scaption 0°–120°—with internal rotation 0°–40°; and external rotation 40°–0° being the other two movements studied. Additionally, Table 1 also includes the peak muscle activation, as output by the OpenSim® simulation suite.

**Table 1:** Maximal muscle forces and peak joint reaction forces (JRF) in different ranges of motion for the natural shoulder model, the reverse shoulder prosthesis model and the dual-bearing shoulder prosthesis model

	Natural shoulder (NS)		Reverse shoulder prosthesis (RSP)			Dual-bearing shoulder prosthesis (DBSP)		
	Force (N)	Activation	Force (N)	% Change from NS	Activation	Force (N)	% Change from NS	Activation
*ABD*								
Anterior deltoid	136.3	0.27	121.7	− 11	0.24	116.5	− 15	0.23
Middle deltoid	292.4	0.25	299.4	+ 2	0.25	396.8	+ 36	0.33
Posterior deltoid	330	0.20	156.5	− 53	0.10	107.9	− 67	0.07
Supraspinatus	56.1	0.09	–	–	–	–	–	–
Infraspinatus	95.4	0.07	152.7	+ 60	0.11	130.2	+ 36	0.09
Teres minor	36.6	0.07	118	+ 222	0.24	68.9	+ 88	0.14
Subscapularis	213.6	0.15	205	− 4	0.14	58.6	− 73	0.04
JRF	1054.0		1142.2	+ 8		957.5	− 9	
*SCP*								
Anterior deltoid	126.7	0.25	143.3	13	0.28	146.4	16	0.29
Middle deltoid	227.1	0.19	265.5	17	0.22	370.4	63	0.31
Posterior deltoid	281.8	0.17	195.2	− 31	0.12	137.2	− 51	0.08
Supraspinatus	53.4	0.09	–	–	–	–	–	–
Infraspinatus	220.9	0.15	255.9	16	0.18	259.5	17	0.18
Teres minor	38.6	0.08	139.5	261	0.28	80.7	109	0.16
Subscapularis	40.2	0.03	19.8	− 51	0.01	14.7	− 63	0.01
JRF	1031.6		1176.8	14		1095	6	
*IR*								
Anterior deltoid	110.2	0.22	113.9	3	0.23	130.4	18	0.26
Middle deltoid	187.4	0.16	277.2	48	0.23	307.4	64	0.26
Posterior deltoid	19.2	0.01	30.9	61	0.02	43.1	124	0.03
Supraspinatus	50.2	0.08	–	–	–	–	–	–
Infraspinatus	374.8	0.26	338	− 10	0.24	392.3	5	0.27
Teres minor	180.3	0.36	162.5	− 10	0.33	117.6	− 35	0.24
Subscapularis	110.7	0.08	19.9	− 82	0.01	14.5	− 87	0.01
JRF	827.6		935.9	13		946.3	14	
*ER*								
Anterior deltoid	122.7	0.24	112.4	− 8	0.22	133.7	9	0.26
Middle deltoid	157.8	0.13	292.4	85	0.25	309.9	96	0.26
Posterior deltoid	17.9	0.01	39	118	0.02	44.4	148	0.03
Supraspinatus	49.6	0.08	–	–	–	–	–	–
Infraspinatus	369.3	0.26	329.3	− 11	0.23	392.4	6	0.27
Teres minor	171.3	0.34	151.6	− 12	0.31	108.4	− 37	0.22
Subscapularis	111.6	0.08	16.2	− 85	0.01	14.6	− 87	0.01
JRF	779.5		935	20		982.5	26	

*ABD* abduction, *SCP* scaption (SCP), *IR* internal rotation, *ER* external rotation

### Joint reactions forces

**Fig. 1:**
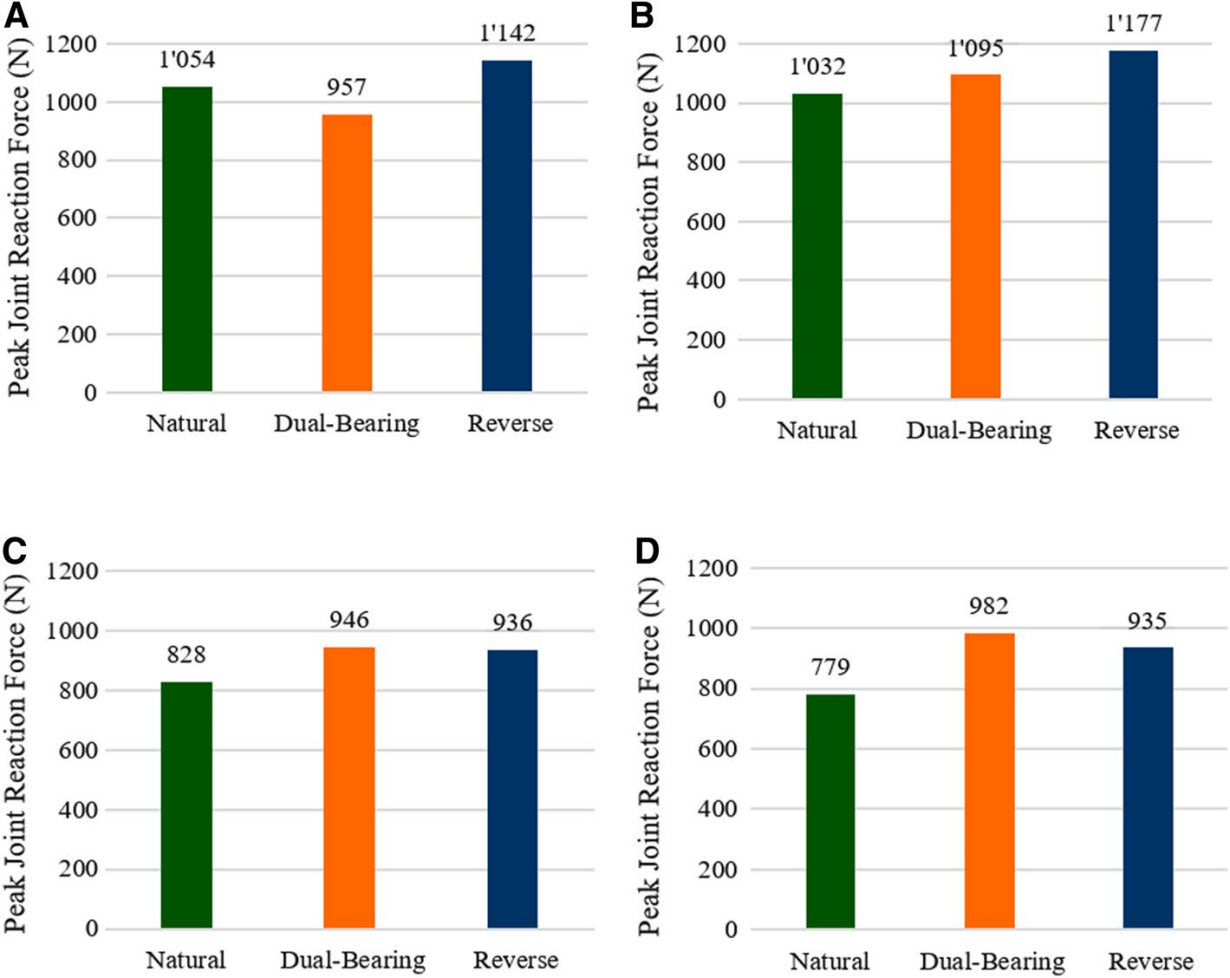
Peak total joint reaction forces for four simulated motions for the natural shoulder, the dual-bearing shoulder and the reverse shoulder prosthesis model for abduction (**A**), scaption (**B**), internal rotation (**C**), and external rotation (**D**)

Considering first, the two large ROM movements, the novel DBSP model generated the lowest joint reaction forces of all the three models during abduction (Fig. 1A). JRF were 10% and almost 20% lower than the NS and RSP models, respectively. For scaption (Fig. 1B), the DBSP JRF were slightly higher (~ 6%) than the NS model, but were lower than the forces generated in the RSP model, which were almost 15% greater than for the natural shoulder (NS). On the other hand, the DBSP model generated the largest JRF for internal and external rotation (Fig. 1C and D). Nevertheless, they were comparable to the RSP model (~ 1% and 5% difference, respectively). Consequently, the differences between DBSP and NS models—12% for internal- and 20% for external rotation—were very similar to the corresponding differences between RSP and NS models (11.5% and 17%, respectively).

### Muscle forces for the different motions

The shoulder muscles investigated fall into two groups: deltoid muscle group consisting of the anterior, middle and posterior deltoids, and the rotator cuff group, which comprised the supraspinatus, infraspinatus teres minor and subscapularis. Individual muscle peak forces are delineated in Table 1.

**Fig. 2:**
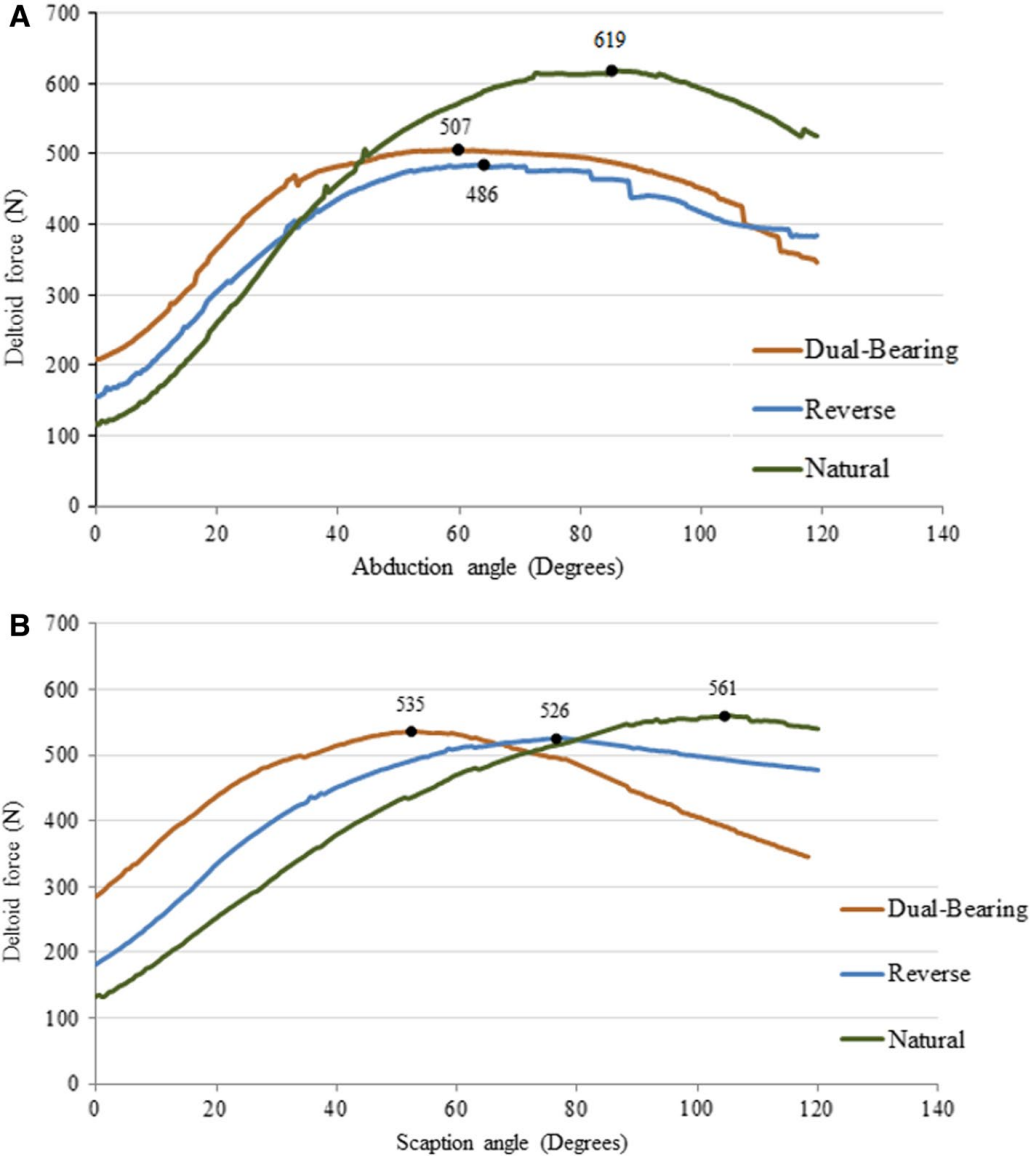
Deltoid muscle force vs abduction angle (**A**) and deltoid muscle force vs scaption angle (**B**) for different shoulder models

**Fig. 3:**
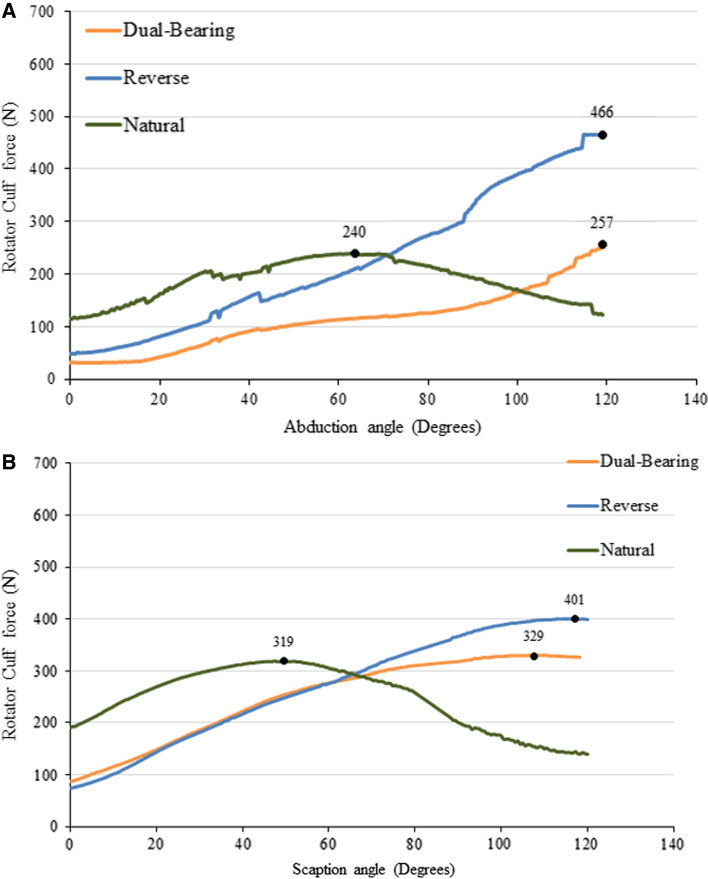
Rotator cuff muscle force vs abduction angle (**A**) and rotator cuff muscle force vs scaption angle (**B**) for different shoulder models

The total deltoid muscle group peak forces in the DBSP model are similar to those generated in the RSP model for abduction (~ 4% difference; Fig. 2A) and scaption (~ 1.7% difference; Fig. 2B)—the two large ROM motions, but 18% and 22% less, respectively, than those generated in the NS model. Differences in rotator cuff muscle forces between the DBSP and NS models were small during simulated abduction (6%; Fig. 3A) and scaption (3%; Fig. 3B). However, rotator cuff muscles elicited a significantly lower effort in the DBSP model compared to the RSP model in both abduction (45%; Fig. 3A) and scaption (22%; Fig. 3B).

For internal and external rotation, the deltoid muscles generated substantially larger forces in the prosthesis models—both DBSP and RSP—compared to the NS model. However, the opposite occurred within the rotator cuff muscles. The biggest reductions compared to the NS model were seen in the subscapularis muscle force for both, DBSP and RSP models—82% and 87%, respectively, for internal rotation, and 85% and 87%, respectively, for external rotation. In external rotation, the second largest reduction (37%) after subscapularis muscle was seen in the teres minor for the DBSP model, compared to a relatively modest 12% reduction in the RSP model.

### Muscle activation

Muscle activation is the ratio of actual muscle force to the maximum isometric force that a muscle can generate. Its value lies between 0 and 1 and it implies the amount of relative effort of the muscle element. For each muscle group, the sum of peak forces produced by each muscle element in that group during a particular motion was divided by the sum of maximal isometric forces of those muscle elements to calculate the peak muscle activation in order to derive the average effort of the muscle system for comparison purpose.

Table 1 clearly shows that the muscle activation for all the models during four different motions is below 0.5, implying a realistic effort simulated by the models. It can be seen that the middle deltoid activation for the DBSP model is higher than for the RSP model. However, the activations for the posterior deltoid and rotator cuff muscles are lower for the DBSP model compared to the RSP model.

## Discussion

Our overall goal was to assess the biomechanical performance of the dual-bearing shoulder prosthesis, which purportedly claims to address limitations in the currently available reverse shoulder prosthesis designs. In this initial study, we adopted an in silico approach, wherein we deployed a validated computational musculoskeletal model of the shoulder joint complex to estimate the gleno-humeral joint reaction forces, and the shoulder muscle forces and activations (muscle effort). We then compared these outputs between models adapted for the natural shoulder, the "conventional" reverse shoulder prosthesis and the "novel" dual-bearing shoulder prosthesis for four common shoulder movements: abduction, scaption, internal and external rotation. This approach enabled a verification of the claims with respect to superior, or, at the very least, equivalent biomechanical performance of the dual-bearing prosthesis compared to the "conventional" reverse shoulder prosthesis. In this regard, the biomechanical performance of the DBSP can be considered to benchmark against RSP, which is the currently available design.

The results confirm the findings from other studies, that the RSP generates lower deltoid muscle forces by increasing the moment arm during abduction compared to the NS [4, 7, 8]. Further validation was also carried out by comparing results for abduction to the results of Bergmann et al. [34] and Costantini et al. [35], see details in the section "Method".

**Fig. 4:**
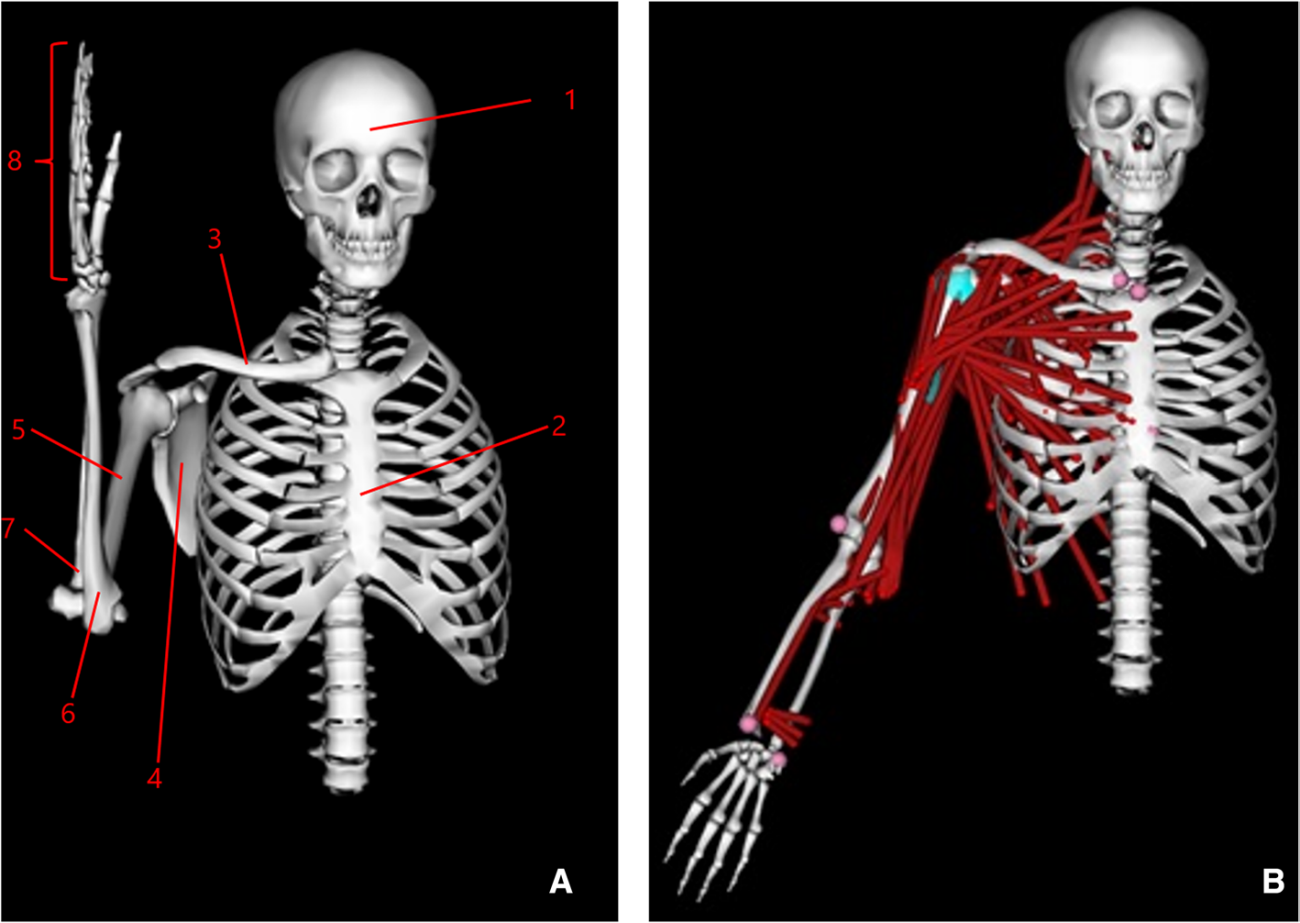
OpenSim® shoulder model incorporating the shoulder joint complex without muscles: head (1), thorax including spine (2), right clavicle (3), right scapula (4), right humerus (5), right ulna (6), right radius (7), and right hand (8) (**A**); OpenSim® shoulder model with all involved muscles (red), markers on bony landmarks (pink) and wrapping surface in humerus for the muscles (blue) at 30° scaption (**B**)

For the DBSP, the peak deltoid muscle forces were lower in abduction compared to the NS model (Fig. 2), in scaption the peak deltoid muscle force was higher during lower scaption angles (0–70°), but lower during higher scaption angles (70°–120°) compared to the NS and the RSP model (Fig. 4). Overall, the difference in peak values of deltoid muscle forces during abduction and scaption between the reverse and dual-bearing designs was not substantial. This indicates that the dual-bearing prosthesis is a viable option biomechanically for shoulder replacement surgery in future.

Not only did the DBSP result in lower JRF than the RSP during abduction and scaption—the two large ROM movements, but these value were either equivalent or lower than those generated in the natural shoulder. Although the forces were much larger than for the natural shoulder during internal and external rotation, they were quite similar to the joint forces developed in the RSP model. Assessing the magnitude of joint reaction forces is useful to understand the risk of implant failure due to dislocation or outright damage on the one hand, but also to assess longer term risks of wear-related complications. In this regard, given that the DBSP model generated lower or equivalent JRF to the RSP model indicate that the DBSP performed quite favorably in comparison to the RSP.

The basic musculoskeletal shoulder model used in this research is a verified one originally built in SIMM [28], incorporating all involved joints from the shoulder: the gleno-humeral joint, the sterno-clavicular joint, the acromio-clavicular joint, and the scapula-thoracic joint [33].

With respect to muscle forces, peak DBSP forces in the deltoid muscle group were similar to those generated in the RSP for all the four movements investigated, again implying an equivalent biomechanical performance in this regard.

The most notable outcome in this study related to the efforts (force and activation) elicited in the rotator cuff muscles for the four movements studied. The DBSP model generated significantly lower forces compared to the model representing the conventional reverse shoulder prosthesis. Furthermore, the subscapularis, which plays a substantial role during internal rotation, also developed significantly lower forces during internal rotation in the DBSP model compared to the NS model, but a much more modest, yet notable reduction compared to the RSP model. Similarly, the teres minor, which has an important role in external rotation, also developed significantly lower forces with the DBSP model compared with both, the natural shoulder and the RSP models. With regard to abduction and scaption, it is generally accepted that the deltoid muscles will generate less force in the prostheses with respect to the natural shoulder. While this certainly held true, the novel DBSP model also revealed results similar to the RSP. This indicates that, under the made assumptions, the new design is better, or, at the very least not inferior to the conventionally available RSP design when assessing demands placed on the shoulder muscles.

Overall, these results indicate that the novel DBSP design can be a feasible alternative for patients suffering from severe rotator cuff arthropathy, following which rotator cuff muscle activity is severely compromised.

The values of the muscle activation reported in Table 1 imply that all of the muscle groups in the three models have generated efforts below 50% of their maximum capacity. Although the middle deltoid forces and the activations for the DBSP model are higher than for the RSP model, the forces and activations for rotator cuff muscles are lower for the DBSP model compared to the RSP model. This implies that the middle deltoid in the DBSP model bears more load during arm motion to reduce forces in the rotator cuff muscles. This finding further supports the earlier statement that DBSP can be used for the treatment of patients with severe rotator cuff tear, since any weakness in the rotator cuff muscle group is compensated by the higher activity and greater force-generation in the middle deltoid muscle.

In reality, the glenoid ring is just supported to the glenoid. However, the acromion, the coracoid process, and the surrounding muscles limit the motion of the ring within the shoulder joint complex.

One shortcoming of this investigation is that the computer simulation predicts the behavior just after implantation and does not consider the effect of healing and muscle relaxation with the time. Furthermore, this study did not take into account patient-specific anatomic differences that may also affect muscle forces and JRF [10]. This limitation is not crucial, as the study was more focused on comparing the biomechanical behavior of the novel prosthesis with the RSP and the NS, therefore additional anatomic variances would have gone beyond the scope of this study, and would have reduced the significance of the afore-mentioned results.

Although preliminary tests on cadavers with inserted dual-bearing prostheses revealed good functionality of the DBSP without dislocation, further tests will need to be conducted to quantify and confirm the stability of the novel design during different movements, which could not be addressed with the computer simulations.

Despite the limitations of a computational study, as mentioned above, it should be noted that the results of this initial step in the evaluation of the DBSP are promising. The results of this study can provide the basis for future investigations, including in vivo studies wherein the kinematics of the shoulder joint containing the novel prosthesis can be recorded using techniques such as motion capture or videometry. This dataset can then be further compared to the performance of the natural shoulder and reverse shoulder prostheses to help gain a better understanding of the behavior of this novel prosthesis in vivo.

With the help of future validation studies and a proper understanding of how to incorporate computer simulation testing in biomechanics, new prostheses and innovative design variants in TSA can be explored and optimized before their use in clinical trials.

## Conclusion

This research presents a biomechanical comparison of generic musculoskeletal shoulder models, including a NS model and two models including a shoulder prosthesis. Furthermore, the study shows that the novel dual-bearing shoulder prosthesis can be a promising option for patients with rotator cuff arthropathy because of its lower rotator cuff muscle forces during all four arm motions compared to the reverse shoulder prosthesis. Peak values of deltoid muscle force for reverse and dual-bearing designs were in a similar range during abduction and scaption. Low peak muscle activation values in all models for anterior, middle and posterior deltoids during the four motions reveals that the muscles were not investing high effort when holding 2 kg weight in the hand. Ultimately, this study demonstrates that computer simulation can be a useful tool in the development phase to assess the effects of prosthesis design on the biomechanics of the human musculoskeletal system.

## Methods

### Model of the natural shoulder (NS)

**Fig. 5:**
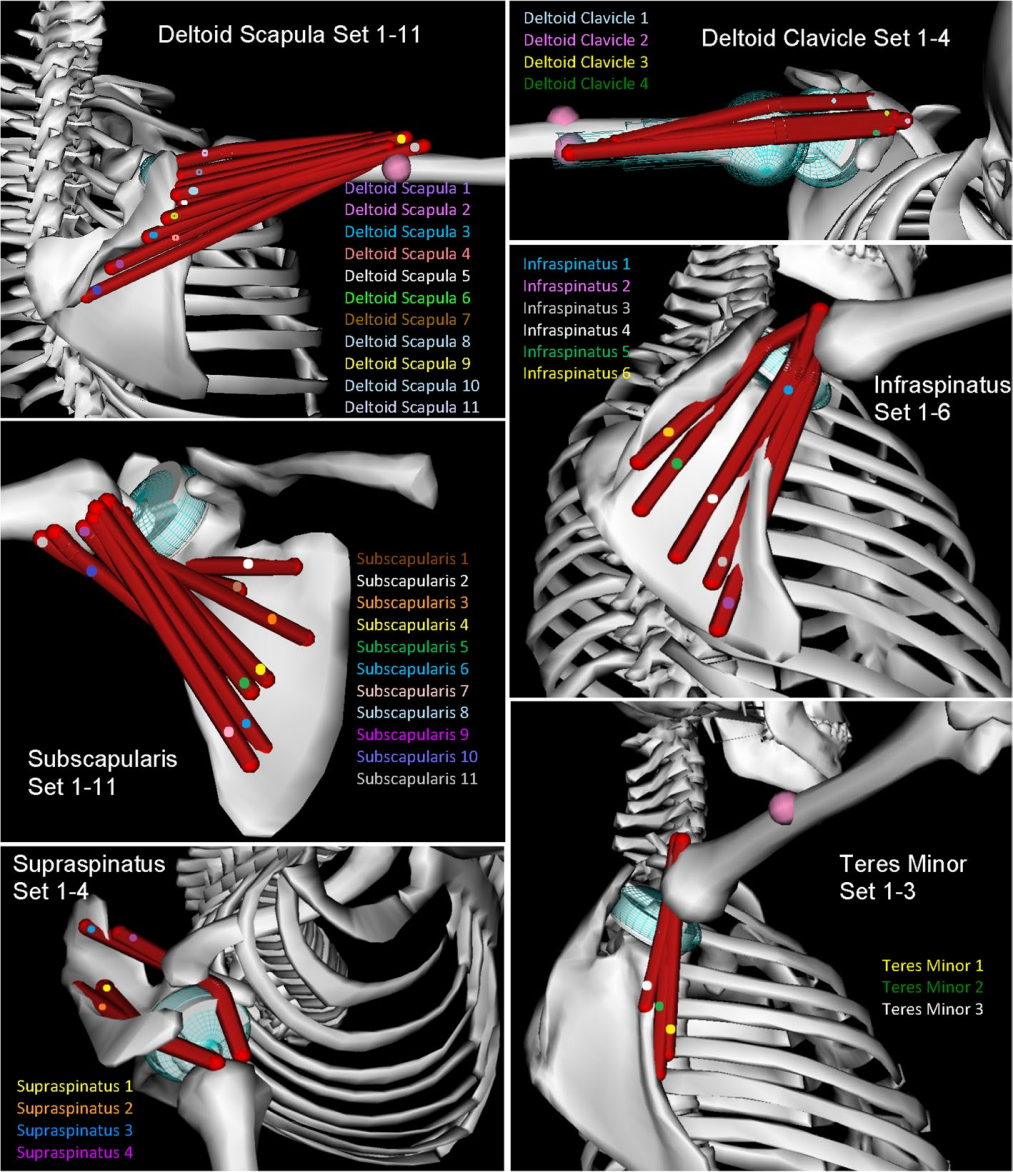
The deltoid and rotator cuff muscle groups acting over the gleno-humeral joint

An existing OpenSim® 3D musculoskeletal model of an upper body incorporating the shoulder joint complex with all involved muscles was used as the NS model in this study (Fig. 4A and B). The model was originally built in SIMM (Software for Interactive Musculoskeletal Modeling, Motion Analysis Corporation, Rohnert Park, California, [27]) by Blana et al. [28], using anatomical data from cadaver studies performed by Klein-Breteler et al. [36]. The well-validated generic model represents the right shoulder of an embalmed 57-year-old muscular man with an estimated height of 168 cm [36]. The model is composed of the following 8 rigid parts: head, thorax including spine, right clavicle, right scapula, right humerus, right ulna, right radius, and right hand (Fig. 4A). It incorporates a gleno-humeral ball–socket joint and a novel scapulo-thoracic joint plugin developed by Seth et al. [33]. The scapulo-thoracic joint plugin provides an improved representation of scapular and clavicular kinematics relative to the thorax during arm motion. The ligaments are not included in the model, as they do not significantly contribute to the joint reaction forces and the corresponding muscle forces [27, 28]. In the whole model, there are 29 muscle groups comprising a total of 138 muscle elements (Fig. 4B). The muscle groups acting over the gleno-humeral joint are shown in Fig. 5. The remaining muscles are listed in Table 2 as given by Blana et al. [28]. Each muscle is assumed as a tensile force-generating element. Muscle forces are calculated based on the Hill muscle model [37]. Major shoulder muscle groups consist of the deltoid group and the rotator cuff group. The deltoid group comprises three subgroups: anterior deltoid (or so-called deltoid clavicle, 4 muscle elements), middle deltoid (4 muscle elements), and posterior deltoid (7 muscle elements). The rotator cuff group comprises four subgroups: subscapularis (11 muscle elements), infraspinatus (6 muscle elements), teres minor (3 muscle elements) and supraspinatus (4 muscle elements). While the supraspinatus is included in the NS model, it is rendered inactive in the models with prosthesis, since this muscle is usually cut by the surgeon in order to enable placement of the implant during arthroplasty surgery. Tendons are implicitly included in the model as they are defined within the muscle model [27].

**Table 2:** Muscles (other than deltoid and rotator cuff muscle group) and the number of elements that represent each one that are included in the models of the DBSP, RSP, and NS

Muscle	Number of elements
Trapezius, scapular part (mid and lower)	11
Trapezius, clavicular part (upper)	2
Levator scapulae	2
Pectoralis minor	4
Rhomboid	5
Serratus anterior	12
Coracobrachialis	3
Teres major	4
Biceps, long head	1
Biceps, short head	2
Triceps, long head	4
Triceps, medial head	5
Triceps, lateral head	5
Latissimus dorsi	6
Pectoralis major, thoracic part	6
Pectoralis major, clavicular part	2
Brachialis	7
Brachioradialis	3
Pronator teres	2
Supinator	5
Pronator quadratus	3
Anconeus	5

To develop the DBSP and RSP models, the NS model was then adapted, wherein the "natural" gleno-humeral joint was replaced by the virtual DBSP or RSP prosthetic joint respectively, as described in the next two sections. As in the original model, no friction between any gliding surfaces was considered in all the three models, reducing the computational time significantly and increasing the numerical stability (convergence of results). This assumption relies on the low coefficient of friction within the natural joint and prostheses (below 0.05 between the UHMWPE and CoCr). An analytical calculation showed that the contribution of friction to the joint reaction moment is below 4% for the DBSP and RSP, see Appendix A. Hence, the contribution of friction is negligible, especially for the present comparison purpose.

### Model including the dual-bearing shoulder prosthesis (DBSP)

**Fig. 6:**
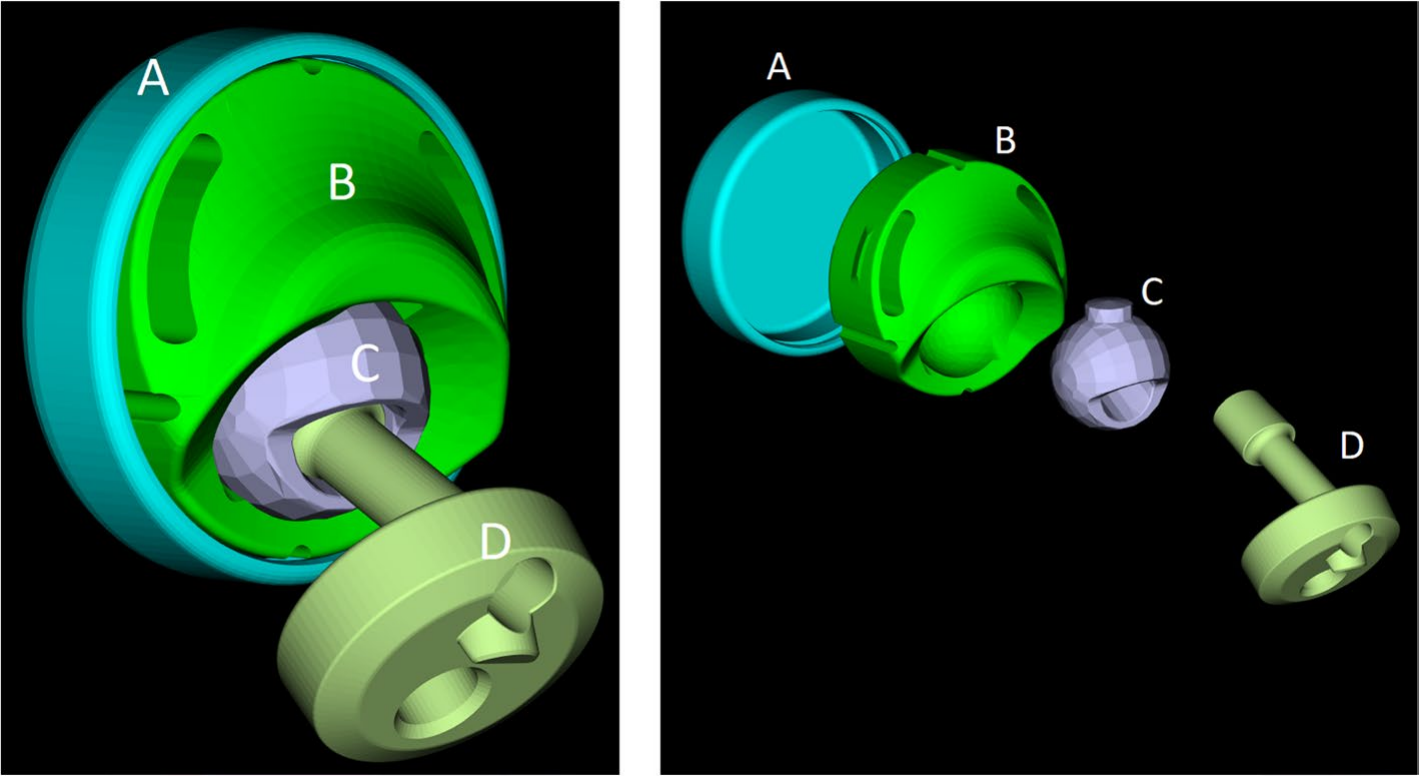
Principal parts of the dual-bearing shoulder prosthesis (DBSP) assembly: glenoid ring (**A**), PE-bearing (**B**); ball head (**C**); and offset adapter (**D**) (left), DBSP shown in an explosion graphic (right)

The dual-bearing shoulder prosthesis concept and design is based on two patents [25, 26]. The prosthesis is assembled by four components and is located between the humerus and the glenoid. For easier reference, the parts are designated with letters A–D, see Fig. 6.

Before being implanted, the ball head (C) is mounted in the PE-bearing (B). Then, the B–C assembly is inserted in the glenoid ring (A). A snap-in system avoids the unlocking between the B and A parts. The offset adapter (D) is then connected to the part C by press-fitting and secured with a screw. Finally, the A–B–C–D assembly is fixed to the humerus stem by press-fitting and secured with a screw and is supported proximally to the following bodies: medially by the glenoid (lateral region of the scapula, which normally supports the humerus head), superiorly by the acromion and anteriorly by the coracoid. Although A and C parts are made of cobalt–chromium (CoCr) alloy, part B of polyethylene, and part D and the humerus stem of titanium (Ti) alloy, all components are assumed as rigid bodies in the model. Part B has a hinge joint in respect to part A which means that part B rotates about the central axis of part A. Part C is connected to part B with restriction due to the engagement of the ball head protrusion in the groove of part B. Furthermore, part C can also rotate about the central axis of its protrusion. Thus, the connection between part C and part B represents a gimbal joint. The scapula–A, C–D and D–humerus stem connections are assumed as welded together (fixed joint) in the model. The fixed joint between scapula and part A allows the analysis of the joint reaction forces (JRF) between scapula and glenoid ring in OpenSim®. In reality, the DBSP does not require glenoid component fixation, it is placed without anchorage. The stabilization is ensured by the acromion, the coracoid process, and the muscles.

The prosthesis system possesses two CORs, one of the joints between the PE-bearing (B) and the glenoid ring (A) and the other between the ball head (C) and the PE-bearing (B). Both CORs are offset from each other. The connections between the parts of the prosthesis and between the prosthesis and the skeleton in the dual-bearing model are summarized in Table 3.

**Table 3:** Joint type and connections in the dual-bearing shoulder prosthesis (DBSP) model

Connecting parts		Type of joint	Degree of freedom (DOF)
Parent	Child		
Scapula	Glenoid disk	Fixed joint	0
Glenoid disk	PE-bearing (inlay)	Hinge joint	1 rotational
PE-bearing (inlay)	Ball head with a protrusion	Gimbal joint	2 rotational
Ball head with a protrusion	Offset adapter	Fixed joint	0
Offset adapter	Humerus	Fixed joint	0

*DOF* degrees of freedom, *PE* polyethylene

**Fig. 7:**
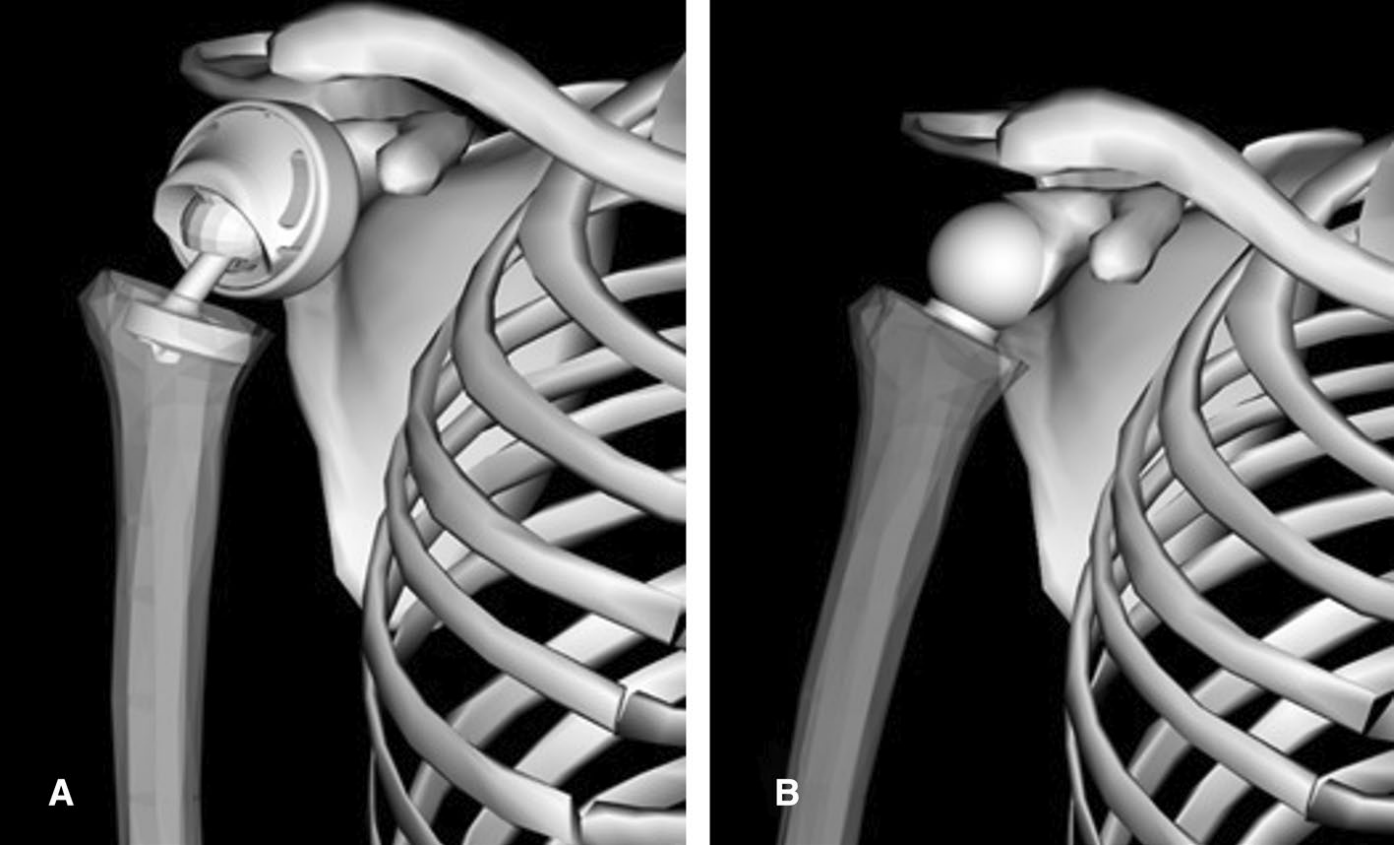
Dual-bearing shoulder prosthesis integrated in the model (**A**), reverse shoulder prosthesis integrated in the model (**B**)

The components of the implant are produced in different sizes in order to allow a best fit with the patient’s anatomy. For this comparative study, the prosthesis with a 55 mm outer diameter of the glenoid ring was chosen with a medium-sized (M) offset adapter. An ellipsoid wrapping surface with principle axes diameters of 75 mm, 75 mm and 28 mm was used in the model to avoid a penetration of the muscles into the DBSP. The wrapping surface provides a virtual deflection support for the middle line of the muscle. In order to deflect the deltoid muscle elements realistically, the diameter of the implant wrap surface was increased by half of the thickness of deltoid muscle elements (estimated to 20 mm). The glenoid ring was fixed to the glenoid fossa and tilted 10° downwards to the glenoid plane (Fig. 7A), similar to the RSP.

### Model including the reverse shoulder prosthesis (RSP)

Basically, the model is built up from a ball-and-socket joint. The RSP was kept in the recommended position for surgery [38–40] in the model as shown in Fig. 7B. A humeral cup was positioned at a suitable angle to the humeral shaft axis in such a way that the prosthesis and other segments do not interfere each other. The COR of the glenosphere was placed in the anteroposterior midline of the glenoid face, 18 mm above the inferior edge of the glenoid rim. As the real glenosphere of hemispherical shape had a diameter of 36 mm, a spherical wrapping surface with a diameter of 56 mm was used for this implant in the model, again considering the deltoid muscle thickness for realistic deflection of the muscle, similar to the model including the DBSP.

### Simulated motions

For the three models, four shoulder motions were simulated: abduction 0°–120°, scaption 0°–120°, internal rotation 0°–40° and external rotation 0°–40°. The inverse kinematics (IK) approach was used to derive rotational joint kinematics from anatomical surface marker-based motion capture data of a single subject, named subject S4, from the shoulder movement database available on SimTK.org [41]. The motions of the markers applied on the subject were measured at 100 Hz. The used markers from the motion capture were duplicated as landmarks in the OpenSim® (V 4.0) model. The motion capture data were then used to drive the model in order to replicate the four shoulder motions. The static optimization (SO) method was applied to obtain muscle forces for individual muscle elements for the given shoulder motion. In the SO method, the muscle forces are resolved by minimizing the sum of squared muscle activations, which are obtained based on muscle–tendon length–velocity dynamics at the particular time point during the simulation movement [27]. The IK motion results were noisy with sudden jumps, which resulted in unrealistic accelerations and constraint violation. Therefore, the SO solver failed to solve the equation for the muscle forces. IK results could have been filtered before using the SO solver to overcome the afore-mentioned problem. However, data filtering could have also altered the marker dataset and the filtered marker positions may differ from the real marker positions. Therefore, in order to achieve convergence of the results, the time scale of the original dataset was stretched by a factor of 1000. In that way, the sudden acceleration jumps/noise present in the data were reduced by the same factor, without the need to filter the original data. Hence, each motion used to calculate muscle forces and JRFs in all the models resembles a quasi-static motion. No inertial forces were considered.

All three models used the same basic set of muscles. The main physical muscle parameters, like the maximum isometric force, optimal fiber length, tendon slack length and the location of the insertion and origin points of the muscles, were taken unchanged from the NS model. Actuators were added to the thorax in all the models to better follow the driven motion. Elbow and wrist were kept fixed during the simulations. The supraspinatus muscle was deactivated during simulation on RSP and DBSP models, considering that supraspinatus is usually impaired during surgery.

Besides the self-weight of the body parts, a weight of 2 kg rigidly attached to the hand, simulating a held object, was defined for the simulation of the different arm motions in each of the three models. The starting position of IR and ER is the palm of the hand looking to posterior.

### Validation of the natural shoulder (NS) model

In order to validate our model, the natural shoulder (NS) model simulation results for abduction motion were compared to a unique experimental study by Bergmann et al. Second, JRF results for abduction and scaption motion from our reverse shoulder prosthesis model simulation were also compared to results reported by Constantini et al. [35] for the Newcastle reverse shoulder model. The shoulder joint reaction forces were calculated for the NS model with 2 kg load in the hand and compared with the in vivo gleno-humeral joint loads measured on patients using an instrumented shoulder prosthesis [34, 42]. The maximal JRF at 90° abduction for the NS model amounts 1054 N, which is 9.9% higher than reported by Bergmann et al. [34] for slow motions. Notice, that the Bergmann's study shows a strong variability from subject to subject. The JRF of our NS model is in the mid-range (i.e., 143%) of the results of the study varying between 69%BW and 188%BW.

Besides this, joint reaction forces during abduction and scaption for the Newcastle reverse shoulder model used by Costantini et al. [18] and the reverse shoulder model used in this study were also found in a similar range for different lateral positions of the implant.

## Figures and Tables

**Fig. 8:**
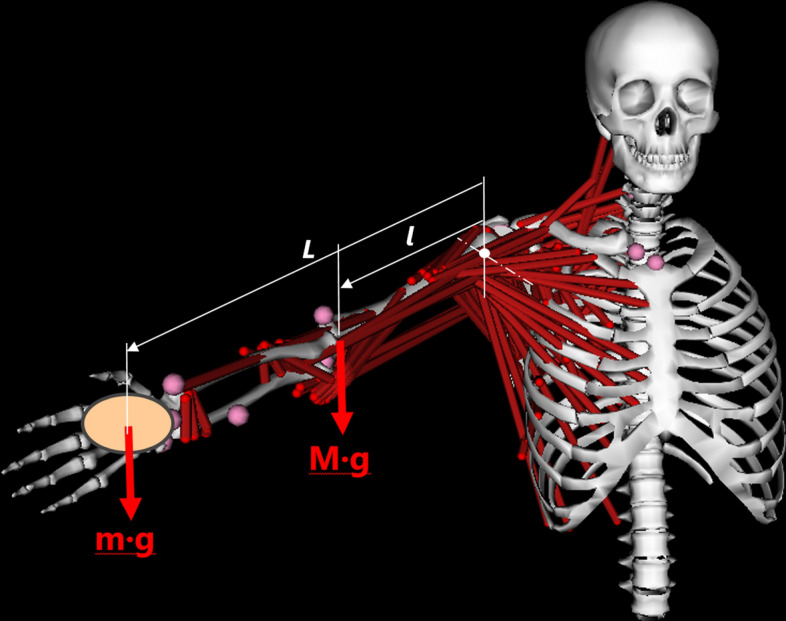
OpenSim® shoulder model used for the estimation of the contribution of the friction on the total moment around the gleno-humeral joint and the prosthesis COR, respectively. The axis around which the moment is calculated for abduction is shown with dash-dotted line, the arm is in horizontal position (90°).

## Data Availability

Not applicable/the generated models of the current study are available in the OpenSim repository.

## Appendix A

The considered material combination and surface finish used for the DBSP ensure a low friction of about μ = 0.05 (UHMWPE against CoCr alloy). The maximal JRF in the COR of the prosthesis in abduction amounts to approximately 1000 N, thus the contribution of friction to the moment acting around the COR of the prosthesis is:

M_f_ =  μ * JRF * r,

with μ friction UHMWPE–CoCr alloy (0.05); JRF: joint reaction force (~ 1000 N, see Table 1); r: moment arm for the friction force which is equal to the radius of the prosthesis (DBSP: 12.5 mm, RSP: 18 mm); M_f DBSP_ = 0.05 * 1000 * 12.5 = 625 Nmm or 0.625 Nm; M_f RSP_ = 0.05 * 1000 * 18 = 900 Nmm or 0.900 Nm.

The moment arising from the weight of the arm and the 2 kg weight in the hand is (Fig. 8):

M_ext_ = M*g*l + m*g*L = 4.275 * 9.81 * 0.3 + 2 * 9.81 * 0.6 = 24.35 Nm,

with M: mass of arm (4.275 kg, see [42]); m: mass in the hand (2 kg); l: moment arm of the arm weight (~ 0.3 m); L: moment arm of the hand (~ 0.6 m); g: gravity (9.81 m/s^2^); M_ext_ is independent of the prosthesis type.

The contribution of the friction C_f_ in the total moment acting around the COR of the corresponding prosthesis is:

C_f DBSP_ = M_f DBSP_ / M_ext_ = 0.625 Nm / 24.35 Nm = 2.6%,

C_f RSP_ = M_f RSP_ / M_ext_ = 0.900 Nm / 24.35 Nm = 3.7%.

The contribution of friction to the joint reaction moment is < 4% for both prostheses, the DBSP and RSP, thus its contribution can be neglected.

## Abbreviations

ASP: Anatomical shoulder prosthesis
BW: Body weight
COR: Center of rotation
DBSP: Dual-bearing shoulder prosthesis
DOF: Degree of freedom
IK: Inverse kinematics
JRF: Joint reaction force
NS: Natural shoulder
PE: Polyethylene
ROM: Range of motion
RSP: Reverse shoulder prosthesis
RTSA: Reverse total shoulder arthroplasty
SO: Static optimization
SIMM: Software for Interactive Musculoskeletal Modeling
TSA: Total shoulder arthroplasty
